# *Burkholderia cepacia* Sepsis in a Previously Healthy Full-Term Infant

**DOI:** 10.1155/2020/8852847

**Published:** 2020-10-05

**Authors:** Carlos A. Carmona, Alberto Marante, Fatma Levent, Sarah Marsicek

**Affiliations:** ^1^Pediatric Residency, AdventHealth for Children, Orlando, FL 32803, USA; ^2^Department of Pediatric Intensive Care, AdventHealth for Children, Orlando, FL 32803, USA; ^3^Department of Pediatric Infectious Diseases, AdventHealth for Children, Orlando, FL 32806, USA; ^4^Department of Pediatric Hospital Medicine, AdventHealth for Children, Orlando, FL 32803, USA

## Abstract

*Burkholderia cepacia* causes sepsis in neonates who are immunocompromised or exposed via nosocomial transmission. We report a case of *B. cepacia* sepsis in a previously healthy 5-week-old male originally treated for bacterial pneumonia per chest X-ray findings and 3 days of fevers. Regardless of appropriate antibiotics and an initial negative blood culture, he developed severe hypoglycemia, circulatory collapse with disseminated intravascular coagulopathy, and expired. A second blood culture taken following transfer to the intensive care unit resulted positive for *B. cepacia* postmortem. Review of the newborn screen and family history was otherwise normal. Subsequent postmortem autopsy showed multifocal bilateral pneumonia with necrotizing granulomatous and suppurative portions of lung tissue. Additionally, there was a prominent cavitary lesion 2.5 cm in the right lower lobe with branching and septate fungal hyphae. Stellate microabscesses with granulomas were present in the liver and spleen. These findings plus *B. cepacia* bacteremia are highly suggestive of an immunocompromised status. Review of the literature shows that its presence has been associated with chronic granulomatous disease. Therefore, in a persistently febrile infant not responding to antibiotics for common microbes causing community-acquired pneumonia, immunodeficiency workup should ensue in addition to respective testing for chronic granulomatous disease especially if *B. cepacia* culture-positive as it is strongly associated with neutrophil dysfunction.

## 1. Introduction

*B. cepacia* (*Bcc*) is an aerobic, catalase-producing, Gram-negative bacillus not considered part of normal human flora but typically of low virulence. It can severely affect immunocompromised children such as those with malignancy, congenital heart disease, or a history of prematurity. Pediatric cases describe severe bacteremia in children with cystic fibrosis (CF) and chronic granulomatous disease (CGD) [1].

Immunocompetent individuals are not commonly affected; however, nosocomial infections have been observed due to contaminated medications, fluids, antiseptics, and medical equipment. Examples include contaminated sodium chloride, distilled water, 5% dextrose, and even ultrasound gel. Nebulization, flushing orogastric tubes, and humidification of oxygen delivery devices are the vehicles of transmission [2, 3].

Two of the earliest known case reports involving *Bcc* sepsis in neonates were in preterm infants. One was an ex-24-weeker with extremely low birth weight who developed thrombocytopenia to 26,000 at 24 days of life and despite ceftazidime + imipenem, died from an ileocutaneous fistula and necrotizing enterocolitis. The other patient was an ex-22-weeker who grew *Candida albicans* and was treated with amphotericin and flucytosine, and by day 62 of life, the patient had thrombocytopenia. A positive culture for *Bcc* was resulted, and ceftazidime appropriately cleared the infection [2]. Patra el al. described a cohort of 12 neonates in India whose gestation ranged from 29 to 41 weeks and primarily had lethargy, tachypnea, or poor feeding. *Bcc* was isolated from blood cultures, and neonates were treated with piperacillin-tazobactam, ciprofloxacin, and cotrimoxazole either singly or in combination to result in an eventual sterile repeat culture [4]. Chandrasekaran et al. identified a group of 59 average full-term neonates in India where most (59%) had *Bcc* early-onset sepsis with predominantly respiratory, hemodynamic instability, and abdominal distension. Over 95% either had a previous peripheral IV line used or IV antibiotics administered, and only 29% had maternal risk factors. Piperacillin-tazobactam was the empirical first-line antibiotic [5].

## 2. Case Presentation

We describe a case of *Bcc* sepsis in a previously well 5-week-old ex-full-term infant. He was admitted to an outlying hospital for fever of one day duration. After two days of antibiotic treatment with minimal improvement, he was transferred to our hospital for further management.

He was delivered by cesarean section due to failure to progress at 39 weeks of gestation. The mother was a 22-year-old Hispanic woman gravida 1 para 1 with an unremarkable prenatal history including a negative group-B *Streptococcus* screen. The infant had respiratory distress after birth requiring continuous positive-pressure support and oxygen for a short duration. Sepsis evaluation at that time was negative, and he was discharged home after a two-day hospital stay. He did well at home with no exposures to daycare, animals, or travel outside the country. No family history of chronic disorders, recurrent infections, or known immunodeficiency was found.

**Figure 1:**
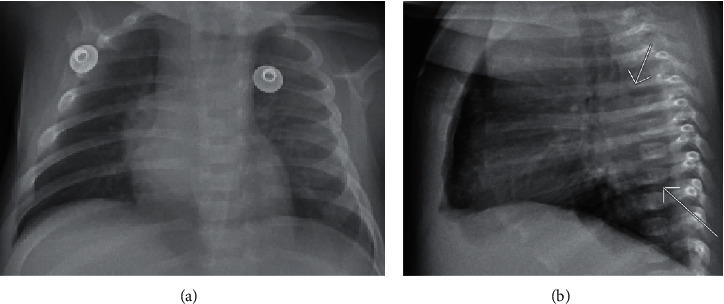
(a) Chest radiograph showing focal airspace opacity projecting over the right midlung zone. (b) Lateral CXR of “well-defined right lower lobe opacity interpreted as pneumonia versus mass.”

At the outlying hospital, the review of systems was significant for decreased appetite. Rhinorrhea and sneezing preceded 1.5 weeks prior to the ER visit. Vital signs recorded a temperature of 102.7°F, respiratory rate of 45 breaths/min, and pulse of 190 beats/min. Physical exam revealed a well-appearing infant with normal cardiopulmonary and abdominal exams but with a faint erythematous maculopapular rash throughout the chest and legs. There was no stomatitis, perianal abscesses, or other signs of skin infections. Laboratory evaluations showed a white blood cell count of 10,000 *μ*L and a band count (16.5%). Chest radiograph showed “focal airspace opacity projecting over the right midlung zone, probably pneumonia” (Figure 1(a)). Ampicillin and gentamicin were started after full sepsis workup was performed. Blood and urine cultures were negative after 48 hours. Respiratory viral panel including influenza and respiratory syncytial viruses were also negative. Cerebrospinal fluid (CSF) was described as cloudy and red with a red blood cell count of 95,880 mm^3^ and WBC count of 170 mm^3^ with lymphocytic predominance (90%). CSF protein level was 114 mg/dL, and glucose level was 58 mg/dL; CSF studies were equivocal due to a grossly hemorrhagic tap. Ceftriaxone (at meningitic dosing) and intravenous acyclovir were initiated. Follow-up meningoencephalitis polymerase chain reaction panel including herpes simplex virus 1 and 2, and cultures were all negative. Subsequent chest radiograph showed “well-defined right lower lobe opacity interpreted as pneumonia versus mass” (Figure 1(b)). The latter prompted an abdominal ultrasound which was essentially negative.

During the first four days in our hospital, the infant continued to be febrile but had an overall reassuring downtrending fever curve. He looked well clinically, but C-reactive protein (179.1 to 247 mg/L) and band count (26% to 44%) increased by day four to five of hospitalization when his condition abruptly deteriorated. The patient developed hypoglycemia and subsequent pulseless electrical activity, required cardiopulmonary resuscitation, and endotracheal intubation prompting immediate transfer to the pediatric intensive care unit (PICU). Antibiotic therapy was broadened to vancomycin, cefepime, and metronidazole.

He developed disseminated intravascular coagulopathy (DIC) which was not responsive to aggressive resuscitation with packed red blood cells, fresh frozen plasma, cryoprecipitate, and platelets. Cardiac contractility was poor prompting epinephrine and milrinone infusions to improve cardiac output. He had three episodes of cardiovascular collapse that required aggressive resuscitation efforts but succumbed at the third event. It was afterwards that the repeat blood cultures performed at the time of central venous line insertion on PICU admission subsequently resulted positive for *Bcc*.

Autopsy revealed bilateral pneumonia consisting of mixed acute and necrotizing granulomatous inflammation with suppurative foci. The largest area of consolidation was in the right lower lobe with a cavitary 2.5 cm lesion demonstrating septate and branching fungal hyphae. This finding of fungal hyphae within the cavitary lesion likely indicated a coinfection. No viral cytopathic changes were visualized. Hepatomegaly and splenomegaly were identified with areas of acute and granulomatous necrotizing inflammation. Because of the rapid downhill course, no immunological tests were done before death such as immunoglobulin or T cell subsets. No neutrophil oxidative burst or dihydrorhodamine assays were performed. Further genetic testing was advised to the family. Even though these tests were not performed, CGD remained as the most probable cause of this patient's multiorgan granulomatous disorder. There were no other associated pathogens identified or other probable disorders based on history and autopsy findings to explain the granulomatous findings. What is known is that defects in the oxidative burst can lead to severe infections particularly *Staphylococcus aureus*, *Aspergillus* species, and *Bcc* [6].

## 3. Discussion

*Bcc* is a rare cause of sepsis in newborns, and its transmission involves human contact with heavily contaminated medical devices and disinfectants. It most commonly presents with respiratory tract, urinary tract, and blood stream infections [4]. Of the few reports describing *Bcc* sepsis in neonates, the prenatal course is typically significant for certain hospital exposures or family history of an immunodeficiency. There are risk factors like prematurity, surgeries, or instrumentation [3]. Contributing maternal risk factors such as poor intrapartum or postnatal infection control practices are also noted [7]. Our patient did not have any risk factors such as prolonged mechanical ventilation, chronic nebulized treatments, multiple bronchoscopies, or being on previous antibiotics [3]. His postnatal course consisted of needing some continuous positive airway pressure momentarily without having to go to the neonatal intensive care unit.

The chief initial presentation of fevers and congestion were unusual and not reported as most present with respiratory distress, lethargy, and/or emesis [4]. Although less likely, a nosocomial infection could not be completely excluded. After discharge home, the patient had one visit to the hospital two weeks prior to presenting with fevers for an abdominal X-ray which only showed mildly distended loops of bowel with stool throughout. Aside from this exposure, the patient may have acquired the infection via the nosocomial route from being transported from one hospital to another. Even though *Bcc* has an incubation period of 1–21 days [8], this is less likely considering the patient already presented to the first ER with a right lower lobe opacity.

Even without respiratory distress or hypoxia, pneumonia was the leading diagnosis as the opacity appeared to improve with antibiotics. Neuroblastoma was suspected; however, homovanillic and vanillylmandelic acid urine studies were normal. Congenital cardiac defects were unlikely based on a normal echocardiogram. Absent pulmonary cystic structures or active respiratory distress made congenital pulmonary airway malformation or bronchopulmonary sequestration unlikely. Immunodeficiencies like CF or a hemoglobinopathy were unlikely given the normal infant screen. CGD was never suspected since patients typically have recurrent, life-threatening bacterial and fungal infections in the first year of life [9], and the median age of diagnosis is 2-3 years [10]. Additionally, there were no findings on physical exam Also, the patient did not possess skin abscesses, have a history of otitis media, or was considered failure to thrive. Family also reported a healthy background without immunodeficiency.

Microbial diagnosis for *Bcc* is made following blood culture collection using *Bcc*-selective agar, *Pseudomonas cepacia* agar, or oxidation-fermentation polymyxin bacitracin lactose agar. *Bcc*-selective agar is superior to others as it enhances the growth of *Bcc* while suppressing the growth of other organisms [11]. Overall, *Bcc* is difficult to culture, can initially be negative, and, once collected, can prove challenging to properly identify [2, 9, 12]. This was seen with our patient as the first blood culture taken at the outside hospital on admission was negative despite the patient's symptoms of being febrile. Inappropriate microbial expertise in preparing the blood sample in the specific agars may have resulted in a false negative the first time around.

For the second set of blood cultures taken from the intensive care unit, the susceptibilities for *Bcc* resulted postmortem for levofloxacin, ceftazidime, and cefepime to name a few (Table 1). Unfortunately, the patient had been on ceftriaxone, metronidazole, and vancomycin, and so, the antibiotics could not be further tailored to improve coverage. Sputum cultures resulted positive +1 for *Bcc*, *Stenotrophomonas maltophilia*, and *Enterobacter cloacae* complex, respectively, which were listed in Table 2.

Even though our patient did not have formal immunologic testing done due to rapid patient decline, there are many findings to strongly suggest that the patient had CGD which go beyond pure speculation. To name a few examples, there were the autopsy findings of a widespread granulomatous process with *Bcc* and coinfection with fungal elements in addition to positive blood and sputum cultures. There was a consistent presentation with what is described in the literature of a patient with *Bcc* who had CGD with a primary presentation of pneumonia (being the most common site of infection) followed by lymphadenitis, subcutaneous abscess, liver abscess, and sepsis [10]. The patient's end of his life mirrored previous case reports of newly diagnosed CGD in patients with *Bcc* sepsis. Lacy et al. [12] described findings including hepatomegaly, splenomegaly, ascites, disseminated intravascular coagulation, and granulomas within the lungs, liver, and spleen. The autopsy report not only confirmed the granulomas however made mention of hepatosplenomegaly, scattered microthrombi in multiple organs, and a collection of ascitic fluid upon starting the exam.

With these findings and concerns for their future health and offspring, the parents were advised to seek genetic testing for oxidative burst activity. This would assist with future family planning and determining if other family members had to be evaluated. Testing is critical because mutations can be either X-linked or autosomal recessive; individuals may unknowingly have a decrease in the percentage of positive stimulated granulocytes compared to healthy controls [10, 12].

In the presence of an infant with a previously healthy background not following the typical course for pneumonia, it is important to have a broad differential diagnosis including immunoglobulin or complement deficiencies. If the patient has findings of pneumonia, sepsis, skin abscess, lymphadenitis, or hepatosplenomegaly, then *Bcc* should be considered and confirmed with appropriate testing. Multiple blood cultures may need to be drawn as this organism is difficult to isolate. If initial broad-spectrum antibiotics like ceftriaxone are not effective, more empiric drugs like ceftazidime or meropenem may be beneficial to initiate until further microbe identification. It may not be a priority to conduct a primary immunodeficiency workup in the midst of a fulminant disease course; however, it must be kept in mind early on as it can help with understanding the pathophysiology of the patient's clinical trajectory.

## Figures and Tables

**Table 1:** Blood culture susceptibilities.

Microreports	Susceptibilities	Specimen	Action list
	A	B	C
1	*Burkholderia cepacia* complex	MIC dilutn	MIC interp
2			
3	Amikacin	≥64	R
4	Cefepime	2	S
5	Ceftazidime	2	S
6	Gentamicin	≥16	R
7	Levofloxacin	1	S
8	Meropenem	≥16	R
9	Piperacillin/tazobactam	≥128	R
10	Tetracycline	4	S
11	Tobramycin	≥16	R
12	Trimethoprim/sulfa	≤20	S

**Table 2:** Sputum culture susceptibilities.

A	B	C	D	E
*Enterobacter cloacae* complex				
	MIC dilutn	MIC interp	—	—
Cefepime	8	L	—	—
Cafoxitin	≥64	R	—	—
Ceftazidime	≥64	R	—	—
Ceftriaxone	≥64	R	—	—
Ciprofloxacin	≤0.25	S	—	—
Gentamicin	≤1	S	—	—
Levofloxacin	≤0.12	S	—	—
Meropenem	1	S	—	—
Piperacillin/tazobactam	≥128	R	—	—
Tetracycline	4	S	—	—
Trimethoprim/sulfa	≤20	S	—	—

*Stenotrophomonas maltophilia*				
	Generic interpretation	Generic interpretation numeric	MIC dilutn	MIC interp
Ceftazidime Etest	S	1	—	—
Levofloxacin	—	—	≤0.12	S
Minocycline Etest	S	1	—	—
Trimethoprim/sulfa	—	—	≤20	S

*Burkholderia cepacia* complex				
	MIC dilutn	MIC interp	—	—
Amikacin	≥64	R	—	—
Cefepime	≤1	S	—	—
Ceftazidime	2	S	—	—
Gentamicin	≥16	R	—	—
Levofloxacin	1	S	—	—
Meropenem	≥16	R	—	—
Piperacillin/tazobactam	≥128	R	—	—
Tetracycline	4	S	—	—
Tobramycin	≥16	R	—	—
Trimethoprim/sulfa	≤20	S	—	—
